# Effect on Microstructure and Magnetic Properties of Nd-Fe-B Magnets Through Grain Boundary Diffusion of Tb and Multi-Component Alloys

**DOI:** 10.3390/ma18040736

**Published:** 2025-02-07

**Authors:** Fei Wang, Mei Wang, Wei-Ming Liu, Peng-Fei Wang, Qian Wang, Yu-Meng Zhang, Zhao-Pu Xu, Wei Li, Xin-De Zhu

**Affiliations:** 1 Key Laboratory for Liquid-Solid Structural Evolution and Processing of Materials, Ministry of Education, Shandong University, Jinan 250061, China; hqwangfei@163.com (F.W.); wangmei@sdu.edu.cn (M.W.); qian.wang@sdu.edu.cn (Q.W.); 2 Yantai Standard and Metrology Inspection and Testing Center, Yantai 264000, China; 13863842589@139.com; 3 Yantai ZhengHai Magnetic Material Co., Ltd., Yantai 264006, China; wangpengfei@zhmag.com (P.-F.W.); zhangyumeng@zhmag.com (Y.-M.Z.); xuzhaopu@zhmag.com (Z.-P.X.); liw@zhmag.com (W.L.); 4 Jianxin Zhao’s Group Co., Ltd., Ningbo 315600, China

**Keywords:** Nd-Fe-B, inherent coercivity, grain boundary diffusion, microstructure

## Abstract

In this study, commercial Nd-Fe-B magnets were utilized as starting materials to investigate the impact of various Tb-containing diffusion sources on the magnetic properties. Tb, Tb_60_Nd_5_Al_30_Ga_5_, and Tb_65_Pr_10_Nd_5_Al_5_Cu_10_Ga_5_ were developed as diffusion sources. After grain boundary diffusion treatment, the magnetic parameters of the magnets were evaluated at 20 °C, 90 °C, and 140 °C. The composition, microstructure, and elemental distributions of the magnets before and after diffusion were examined. It was found that the inherent coercivity of the magnets showed a dramatic increment of 49.4% at 20 °C after diffusion with Tb-containing alloys. The benefits and drawbacks of the designed diffusion sources were thoroughly discussed. Magnets diffused with the Tb_65_Pr_10_Nd_5_Al_5_Cu_10_Ga_5_ source displayed the highest overall performance, generating a thin layer with a grid-like structure at the grain boundaries and a consistent shell structure of Tb around the main phase grains. This work offers a promising alternative in the optimization of Nd-Fe-B magnets.

## 1. Introduction

Rare earth metals are widely used in numerous industries, such as transportation, healthcare, national defense, and communication technology, due to their remarkable optical and magnetic properties, which make them essential in the production of alloys, phosphors, glasses, ceramics, catalysts, and permanent magnets. Among these applications, rare earth permanent magnet motors are of great importance. These motors utilize high-performance Nd-Fe-B permanent magnets to provide a robust magnetic field that improves the efficiency of electric vehicles [1].

In 1983, Sumitomo Special Metals Company in Japan created the third-generation rare earth permanent magnet Nd-Fe-B. Owing to its extraordinarily high magnetic energy, Nd-Fe-B has dominated the permanent magnet market. However, the magnetic performance of Nd-Fe-B at high temperature is poor because of demagnetization, leading to low coercivity [2,3]. Therefore, it is vital to improve the coercivity of Nd-Fe-B magnets. Early researchers added heavy rare earth elements such as Dy and Tb during melting to boost coercivity. However, the integration of heavy rare earths into the primary phase Nd_2_Fe_14_B resulted in the creation of heavy rare earth compounds. The 3d electron spin magnetic moments are antiparallel to the rare earth atom magnetic moments, demonstrating ferrimagnetic coupling [4], which reduces the saturation magnetization (Ms) of the material. Additionally, with the expansion of manufacturing, the rising prices of heavy rare earths greatly increase expenses. Thus, decreasing the utilization of heavy rare earths is a critical challenge.

For sintered Nd-Fe-B magnets, demagnetization is hypothesized to be triggered by the nucleation of reverse magnetic domains at places with low local magnetic anisotropy, such as grain boundaries. This diminished anisotropy can be corrected by grain boundary diffusion (GBD) with heavy rare earth (HRE) elements. HREs selectively deposit along grain boundaries, forming HRE-rich shell layers with larger anisotropy fields, hence improving coercivity [5]. Another cause for the higher coercivity after GBD is the thickening of the grain boundary phase. Under the effect of the concentration gradient, HREs diffuse from the grain boundary into the Nd_2_Fe_14_B phase. Furthermore, the (Nd,HRE)_2_Fe_14_B shell layers reject Nd, making the grain boundary phase thicken and thus increasing the magnetic isolation of hard magnetic grains [6]. Since HRE substitution for Nd is limited to locations near the grain boundary, GBD can maximize HRE utilization. Currently, researchers are concentrating on diffusion sources and heat treatment procedures to further increase HRE utilization efficiency [7].

In the study of diffusion sources, the first group often includes heavy rare earth elements (elements such as Dy, Y, and Tb). Nakamura [8] coated Dy_2_O_3_, DyF_3_, and TbF_3_. It was found that fluoride diffusion was more effective than oxide diffusion. The first type considerably boosts coercivity but has low diffusion efficiency and a high cost. The second group comprises light rare earth alloys such as Pr and Nd. Due to the low melting points, they can moisten the grain boundaries during heat treatment, resulting in continuous and smooth grain boundaries, effectively minimizing magnetic coupling [9]. Furthermore, in light rare earth compounds, the 3d electron spin magnetic moments are oriented parallel to the rare earth atom magnetic moments, resulting in ferromagnetic interaction. Therefore, light rare earth compounds have higher Ms than heavy rare earth compounds. Some researchers have employed Pr-Zn [10], Pr-Cu [11], and Pr-Al-Cu [12] alloys as diffusion sources to boost the coercivity of magnets, reducing irreversible magnetic flux loss, and improving high-temperature performance. The second type moderately boosts coercivity, decreases unfavorable effects on remanence, and is cost-effective. The third category comprises low-melting-point alloys comprising non-rare earth elements, such as Al-Cu, MgO, and ZnO [13]. Through the diffusion of these low-melting-point alloys, grain boundaries are suitably wetted with eliminated defects and uniformly distributed and appropriately thickened grain boundaries, which also weaken ferromagnetic grain borders, lowering intergranular magnetic coupling. The third type is low-cost but has a limited coercivity increase and is not suited for use on its own.

Each of the three types of diffusion sources has its particular advantages and limits. Combined with the above research results, to optimize the balance between the diffusion efficiency and performance of commercial NdFeB magnets, in this study, the diffusion of Tb, Tb_60_Nd_5_Al_30_Ga_5_, and Tb_65_Pr_10_Nd_5_Al_5_Cu_10_Ga_5_ in Nd-Fe-B magnets was explored. Magnets after diffusion were denoted as D1, D2, and D3, respectively. The impact of different types of Tb-containing diffusion sources, including pure metal Tb, low-melting-point Tb alloy, and multi-component low-melting-point Tb alloy, on the magnetic performance of Nd-Fe-B magnets was evaluated. The microstructure and elemental distribution were characterized to study the mechanism of the improved diffusion effects, seeking an appropriate diffusion source for sintered Nd-Fe-B magnets.

## 2. Materials and Methods

The initial magnets utilized in this work were commercial N40H Nd-Fe-B magnets, with a room temperature magnetic performance of Br = 13.28 kGs, Hcj = 18.54 kOe, and BH(max) = 41.98 MGOe. The magnets were machined to dimensions of 15 mm × 8 mm × 6 mm (with the c-axis along the 6 mm direction). The diffusion source alloys were made by a melt spinning process and subsequent ball milling for 90 min to obtain micron-sized powders. The powders were then mixed to make a slurry. After degreasing and cleaning, the magnets were coated with the slurry on the top and bottom surfaces perpendicular to the c-axis. The components and weight gain ratios of the slurry are presented in Table 1. In particular, we controlled the weight gain ratio of Tb during the experiment by controlling the weight gain ratio of the slurry, so as to evaluate the diffusion capacity of the diffusion source more objectively.

According to the phase composition and melting temperature of the NdFeB sample, as well as the experience in actual production, the magnets were treated with a diffusion heat treatment at 900 °C for 10 h and annealed at 500 °C for 2 h at a vacuum of 40 Pa. After diffusion and annealing, the magnets were cleaned and polished for examination. For comparison, the initial magnets without treatment were prepared under the same condition. The magnetic properties of the magnets before and after diffusion were tested using the NIM-62000 permanent magnet precision measurement system at 20 °C, 90 °C, and 140 °C. The phase composition of the magnets was examined using a Rigaku-SmartLab X-ray diffractometer (XRD, D/Max2550VB, Tokyo, Japan). The distribution of the main elements was measured with an electron probe microanalyzer (EPMA, JXA-8530F Plus, Tokyo, Japan).

A field emission scanning electron microscope (FE-SEM, JSM-7800F, Tokyo, Japan) was used for morphological imaging and energy dispersive spectroscopy (EDS) investigation.

## 3. Results and Discussion

### 3.1. Magnetic Properties

**Figure 1:**
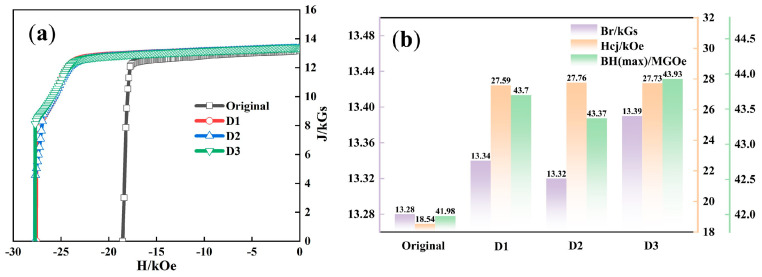
J-H demagnetization curve (**a**) and bar chart (**b**) comparing the magnetic performance before and after magnetic GBD, measured at 20 °C.

The effect of diffusion and annealing was evaluated by measuring the magnetic properties of the magnets. As demonstrated in Figure 1, Hcj greatly improved after diffusion with Tb and its alloys at 20 °C. Given that the weight increase ratio of Tb was consistent, the three diffusion agents had a similar effect on improving Hcj at 20 °C, all reaching over 27.5 kOe, which approximately increased by 49.4% compared to the original magnet. This improvement is related to the greater anisotropy field of the Tb2Fe14B phase created by the Tb element, which provides stronger resistance to demagnetization. Additionally, the diffusion process results in the creation of a Tb-rich shell layer around the primary phase grains, effectively enclosing the grains and lowering the exchange coupling effect of the primary phase grains [14].

The Br of the magnets after diffusion with Tb, Tb_60_Nd_5_Al_30_Ga_5_, and Tb_65_Pr_10_Nd_5_Al_5_Cu_10_Ga_5_ remained around 13.3 kGs in comparison with the original magnet. This suggests that the diffusion of heavy rare earth elements, such as Tb, resulted in limited integration into the main phase, with most residing near the grain boundaries. This is attributed to the much lower saturation magnetization of Tb_2_Fe_14_B than Nd_2_Fe_14_B, as seen in Table 2. Tb_2_Fe_14_B has a much lower saturation magnetization (4πMs = 7.0 kGs) but a very high anisotropy field (HA ~220 kOe) compared with Nd_2_Fe_14_B (4πMs = 16.0 kGs, HA = 73 kOe) and Pr_2_Fe_14_B (4πMs = 15.6 kGs, HA = 75 kOe). This lower 4πMs of Tb_2_Fe_14_B leads to a significant reduction in Br after diffusion. However, the high HA of Tb_2_Fe_14_B significantly enhances the coercivity (Hcj), as a higher anisotropy field promotes stronger resistance to demagnetization.

An excessive amount of Tb entering the main phase would greatly limit Br. Additionally, it was noted that the Br of the magnet diffused with Tb_65_Pr_10_Nd_5_Al_5_Cu_10_Ga_5_ was the highest compared with the magnet treated with Tb and Tb_60_Nd_5_Al_30_Ga_5_, reaching 13.39 kGs. This is due to the fact that the saturation magnetization of Pr_2_Fe_14_B is similar to that of Nd_2_Fe_14_B, partially compensating for the Br loss produced by the development of the Tb-rich shell around the primary phase grains [15].

**Table 2:** Saturation magnetization, magnetic anisotropy, Curie temperature, and lattice constants of Re_2_Fe_14_B [16].

Compound	4πMs (kGs)	HA (kOe)	Tc (K)	a (Å)	c (Å)	ρ (g/cm^3^)
Pr_2_Fe_14_B	15.6	75	565	8.80	12.23	7.54
Nd_2_Fe_14_B	16.0	73	585	8.80	12.20	7.60
Tb_2_Fe_14_B	7.0	~220	620	8.77	12.05	7.96

Regarding the greatest energy product, the original magnet had a BH(max) of 41.98 MGOe at 20 °C. After diffusion, the BH(max) of the magnets treated with Tb, Tb_60_Nd_5_Al_30_Ga_5_, and Tb_65_Pr_10_Nd_5_Al_5_Cu_10_Ga_5_ was 43.70, 43.37, and 43.93 MGOe, respectively.

The BH(max) trend closely mirrors the Br trend. This correlation results from the parameters in the empirical formulas for BH(max) and Br displaying consistent variation trends, as shown in Zhou et al.’s study [4].

(1) $${\left(BH\right)}_{max}=\frac{1}{4}A^{2}\bar{{\cos\theta }^{2}}(1-\beta {)}^{2}{\left(\frac{d_{P}}{d_{T}}\right)}^{2}{\mu_{0}}^{2}{M_{s}}^{2}$$

(2) $$B_{r}=A\;\bar{\cos\theta }\left(1-\beta \right)\left(\frac{d_{P}}{d_{T}}\right)\mu_{0}M_{s}$$

The formula specifies that A represents the volume fraction of aligned magnetic domains, $\bar{\cos\theta }$ denotes the degree of alignment, $\left(1-\beta \right)$ signifies the volume fraction of the main phase, $d_{T}$ stands for the theoretical density of the magnet, $d_{P}$ denotes the actual density, $\mu_{0}$ refers to the permeability of free space, and $M_{s}$ represents saturation magnetization.

In the temperature range of 20–140 °C, it is clear that all magnetic performance indicators for the magnets decrease as the temperature rises. In ferromagnetic materials, there is a direct exchange interaction between the magnetic moments of adjacent atoms, causing them to align in the same direction and manifest magnetic properties. As the temperature rises, the thermal motion of atoms increases, counteracting the exchange energy and reducing the degree of parallel alignment of the atomic magnetic moments, hence decreasing the magnetic properties [4].

**Figure 2:**
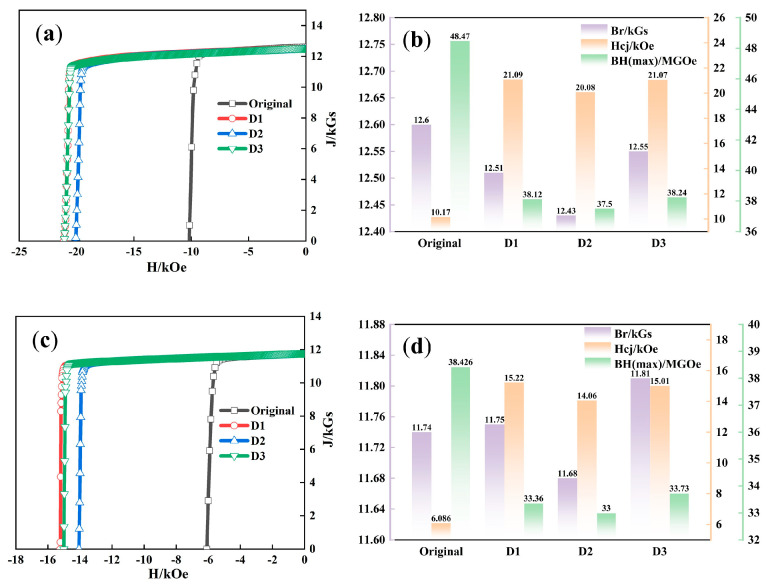
J-H demagnetization curve and bar chart comparing the magnetic performance before and after magnetic GBD at high temperatures: (**a**) 90 °C; (**b**) 90 °C; (**c**) 140 °C; (**d**) 140 °C.

Comparing the magnetic performance of the diffused magnets at high temperature, it is obvious that the magnets subjected to D3 GBD display the best performance (Figure 2). At 90 °C, Br reached 12.55 kGs, with Hcj of 21.07 kOe and BH(max) of 38.24 MGOe. Under an operating temperature of 140 °C, Hcj was 15.01 kOe, slightly lower than the 15.22 kOe of the D1 magnet, while Br and BH(max) were the highest, reaching 11.81 kGs and 33.73 MGOe, respectively.

**Figure 3:**
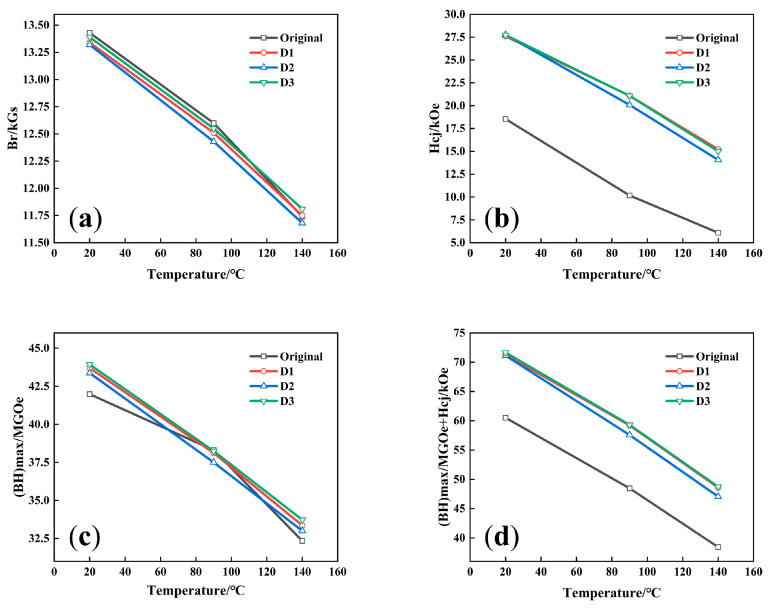
Magnetic properties of magnets at varied temperatures: (**a**) Br; (**b**) Hcj; (**c**) BH(max); (**d**) Hcj + BH(max).

As shown in Figure 3, the curves of Br, Hcj, and BH(max) at different temperatures for each magnet clearly indicate the superior performance of sample D3. In Figure 3b,c, the green line representing sample D3 is positioned at the top among all samples, demonstrating its highest Hcj and BH(max). Additionally, the Br of sample D3 decreases the least with rising temperature, further confirming its optimal comprehensive performance. Figure 3d illustrates the relationship between the sum of BH(max) and Hcj values with temperature. In practical production, this sum is often used to describe the comprehensive performance of magnets. A value exceeding 60 is generally considered indicative of a high-performance magnet. After GBD treatment, the comprehensive performance of sample D3 shows the most significant improvement, with the sum reaching 71.66 at 20 °C, showing the high performance of the magnet.

Table 3 displays the computed temperature coefficients of Br (α) and Hcj (β) for the magnets before and after diffusion. The temperature coefficients of the magnets after diffusion meet the requirements for commercial magnets, which indicate that the Hcj temperature coefficient and the Br temperature coefficient should fall between −0.55 and −0.105%/°C and −0.115 and −0.105%/°C, respectively. It is observed that the D2 sample has poorer temperature coefficients for both Br and Hcj compared to the D1 and D3 samples, indicating deficient temperature stability and a higher likelihood of failure under high environmental temperatures. The Br temperature coefficients of D1 and D3 are essentially equal, and they both have high Hcj temperature coefficients. In this case, the D1 sample performs slightly better, with higher Hcj temperature coefficients in the temperature range, showing that Hcj decreases less with increasing temperature. This improves operational reliability by guaranteeing dependable performance at particular temperatures.

### 3.2. Microstructure and Elemental Distribution

The microstructure and composition of the magnets before and after diffusion were investigated using XRD and EPMA techniques in order to investigate the GBD effect and mechanism in Nd-Fe-B magnets.

**Figure 4:**
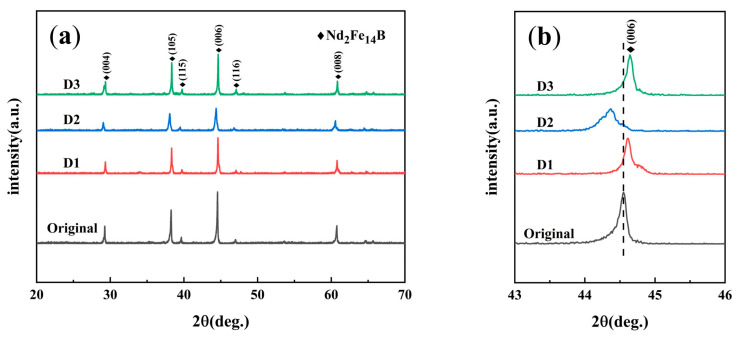
XRD pattern and local magnification (006) of the magnets before and after diffusion (**a**); the enlarged patterns in the range of (**b**) 43–46°.

Figure 4 shows the XRD patterns of the magnets before and after diffusion. It is obvious that the major diffraction peaks of all magnets remain relatively consistent, mostly presenting {001} peaks, which suggests good magnetic orientation [17]. Calculations indicate that the I(006)/I(105) peak intensity ratios for the original, D1, D2, and D3 samples all exceed 1, demonstrating that the c-axis orientation of the magnets was substantially preserved after diffusion [2].

Upon examining the localized magnification of the (006) diffraction peaks, it is observed that the diffraction peaks of magnets D1 and D3 after diffusion exhibit slight shifts toward higher angles. This indicates a reduction in the lattice constants of the main phase, consistent with previous research [15]. The incorporation of Tb into the main phase forms (Nd,Tb)_2_Fe_14_B, which leads to a decrease in lattice constants and causes the diffraction peaks to shift toward higher angles. Conversely, the diffraction peaks of magnet D2 show a shift toward lower angles. This anomalous shift may be attributed to the partial incorporation of Al atoms into the main phase, altering the lattice constants. Given that the diffusion source Tb_60_Nd_5_Al_30_Ga_5_ contains a relatively high Al content, the shift toward lower angles is more pronounced.

**Figure 5:**
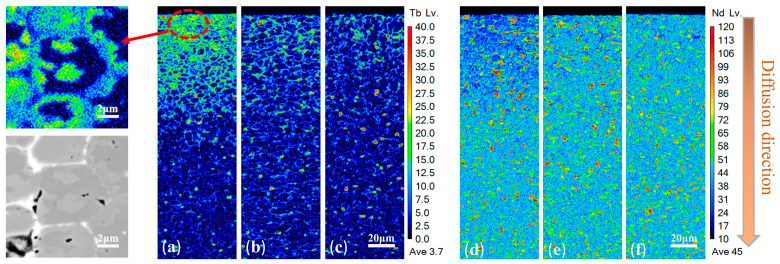
EPMA scans of different diffused samples at cross-sections with a diffusion depth from 0 to 200 μm: (**a**) Tb-D1; (**b**) Tb-D2; (**c**) Tb-D3; (**d**) Nd-D1; (**e**) Nd-D2; (**f**) Nd-D3.

Figure 5 shows EPMA images of D1, D2, and D3 magnet sections at 0–200 µm depth. From the distribution of Tb elements, it can be seen that the Tb diffusion of the D1 sample has an obvious diffusion saturation zone. At the same time, in the diffusion saturation zone of D1, it is observed that there is an anti-shell/core structure, as shown in the red circle in Figure 5, and the Tb content in the grain is higher than that in the shell, which has an undesirable influence on the performance of the magnet. Meanwhile, in the D2 and D3 samples, Tb was mostly found in grid areas near the diffusion surface, indicating that the addition of Al, Ga, and other elements can effectively reduce the formation of a diffusion saturation zone and improve the diffusion efficiency and Tb element utilization efficiency [18]. The grid area in D2 is larger and the grain boundary display is more visible than that of D3, indicating that the grain boundary width is larger.

From the distribution of Nd, it can be seen that there are comparatively few Nd in the diffusion saturation region of the D1 sample with more bulk Nd-rich phases, which also have negative effects on the performance. In the D2 sample, with the diffusion of more elements, the distribution of Nd-rich phases is improved with less bulk Nd-rich phases. The decline in huge Nd-rich phases can also be noticed in the grid region of the D3 sample. This indicates that the design of multi-component diffusion sources with high entropy can effectively optimize the structure of Nd-Fe-B in the diffusion zone while avoiding excessive substitution of Tb for Nd, thus avoiding the appearance of a diffusion saturation zone and increasing the comprehensive performance of the magnet.

**Figure 6:**
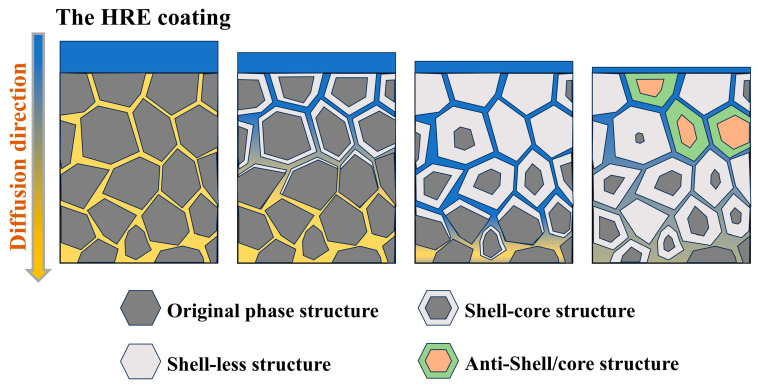
Schematic illustration of the formation mechanism of an anti-shell/core structure.

The formation process of an anti-shell/core structure is shown in Figure 6. At the initial stage of diffusion, Nd in the grains near the surface is replaced by Tb, transforming from the original phase structure to the shell/core structure. At this time, Tb mainly exists in the grain boundaries and the shell layer, and the concentration of Tb at the grain boundaries is higher than that inside the grains. During the middle stage of diffusion, the shell layer keeps thickening, and Tb in the grains near the surface is relatively uniform and has a higher content. There may also be a phenomenon where Nd inside the grains is completely replaced by Tb, that is, the shell-less structure. At this time, the concentration of Tb at the grain boundaries gradually becomes the same as that inside the grains. With the progress of diffusion, the coating is consumed, the content of Tb at the grain boundaries near the surface decreases, and the concentration of Tb inside the grains is higher than that at the grain boundaries, resulting in diffusion from the grains to the grain boundaries to form an anti-shell/core structure [19,20].

**Figure 7:**
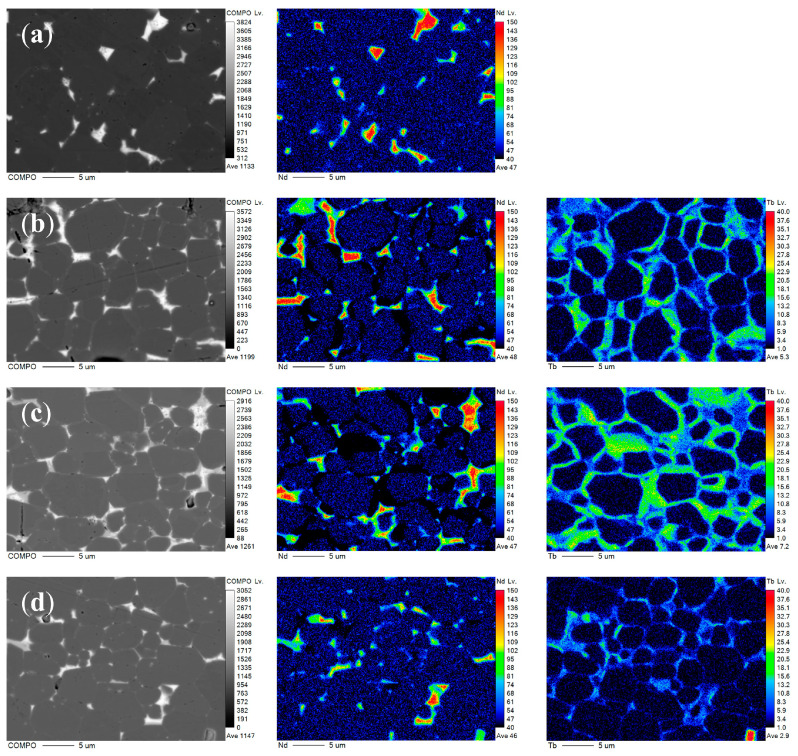
BSE-SEM images and EPMA scans of Nd and Tb of samples before and after diffusion at a diffusion depth of 50 μm: (**a**) original; (**b**) D1; (**c**) D2; (**d**) D3.

To obtain a deeper knowledge of the diffusion mechanism of the three diffusion sources and the differences in the performance enhancements of the magnets, backscattered electron (BSE) pictures and elemental maps of Nd and Tb were obtained using EPMA at a diffusion depth of 50 μm (Figure 7). The same concentration scale was employed across the photos, allowing for easy identification of concentration changes through color variation in the elemental distribution maps.

The brighter areas in the BSE pictures indicate regions rich in rare earth elements. In the BSE picture of the original sample (Figure 7a), the interfaces predominantly consist of grain boundaries between primary phase grains and bulk Nd-rich phases. These boundaries are prone to serve as nucleation sites for demagnetization domains, resulting in easier propagation of magnetic domains and lower Hcj. In contrast, the diffused samples display apparent grain boundaries. The primary phase grains are separated by grid-like grain boundaries, considerably improving the demagnetization resistance and Hcj.

The post-diffusion samples also exhibit an increment in agglomerated Nd-rich phases, indicating the migration of agglomerated Nd-rich phases during the GBD heat treatment, thus optimizing the grain boundary structure [21,22]. Careful inspection reveals a light gray shell around the primary phase grains in the diffused samples, showing that Tb partially infiltrated the main phase, generating (Nd,Tb)_2_Fe_14_B with greater anisotropy fields, contributing to the enhanced Hcj.

As for the diffusion effects of different sources, D1 and D2 showed similar Nd and Tb distributions, with Tb uniformly scattered around the primary phase grains generating shell structures. This considerably boosted Hcj, consistent with experimental results [23]. The presence of Tb at the grain boundaries also influenced the Nd distribution. Grain boundaries were thicker in the Tb_60_Nd_5_Al_30_Ga_5_ diffused sample due to the observation of denser Tb shells and boundaries. Under the same heat treatment conditions, this can be explained by the lower melting temperatures of Al and Ga, which increased diffusivity and flowability [24]. The fact that the performance is not as good as D1, however, indicates that a distinct heat treatment for GBD is required in order to maximize the benefits of various diffusion sources. The D3 sample showed unique Tb and Nd distributions. Tb created shell structures surrounding the main phase grains with lower concentrations, preserving Br and other features of Nd_2_Fe_14_B while also demonstrating higher Hcj compared with the D2 sample. Thin boundary structures boost Hcj better than thick ones, retaining the performance of the hard magnetic phase while lowering intergranular magnetic coupling, making them suitable grain boundary structures [21]. Therefore, the thin border structure of D3 adds to its improved overall performance.

The advantage of multi-component diffusion sources is the reduced amount of Tb in the primary phase, which may be explained thermodynamically [25]. Multi-component diffusion sources have higher entropy and lower Gibbs free energy, making them more stable and less likely to enter the main phase during diffusion. Instead, the components preferentially wet and diffuse at the grain boundaries, increasing the diffusion depth. The Nd distribution is also more uniform, with a considerable reduction in agglomerated Nd-rich phases, boosting the overall performance.

**Figure 8:**
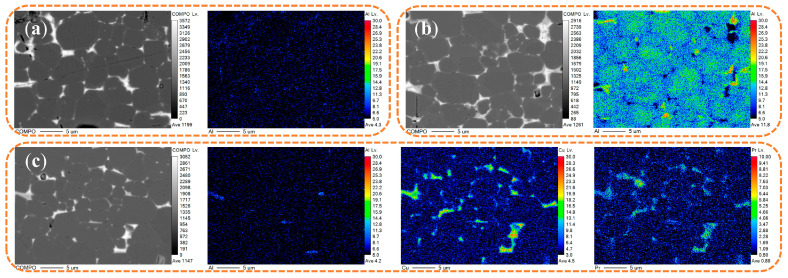
BSE-SEM images and EPMA scan of samples before and after diffusion at a diffusion depth of 50 μm: (**a**) D1; (**b**) D2; (**c**) D3.

EPMA mapping was also undertaken to clarify the roles of Al, Cu, and Pr during diffusion. In Figure 8, the Al distribution is displayed. As a paramagnetic element, Al can reduce the cost of Nd-Fe-B materials and increase the wettability between the primary phase and grain boundary phases, reducing intergranular exchange coupling. However, the high Al in the primary phase of the D2 sample correlates with the sharpest XRD peak shift (Figure 4b), reducing the hard magnetic phase proportion and degrading performance indicators like Br and Hcj compared with D1 and D3. In the D3 sample, Al replaces Fe on grain surfaces, generating the Nd(Fe,Al)2 phase with increased anisotropy, improving Hcj [12]. Some Al enters the Nd-rich phase, boosting the wettability angle between the Nd-rich liquid phase and the primary phase, leading to a more uniform distribution along the borders [26] and greater isolation of the primary phase, further enhancing Hcj.

Copper typically distributes along the grain boundaries. Due to the positive mixing enthalpy between Fe and Cu, Cu cannot substitute for Fe in the Nd_2_Fe_14_B phase [16]. The diffusion of Cu along the grain boundaries lowers the melting point of the Nd-rich phase, improving the driving power for penetration into the magnet [1]. This increases the volume of the grain boundary phase, allowing the grain borders to better encapsulate the main phase grains, hence diminishing magnetic coupling.

In Figure 8c, the distribution of praseodymium (Pr) is comparable to that of Nd. The design of the diffusion source composition seeks to compensate for the drop in Br induced by Tb diffusion and raise the entropy value by adding components. Pr is equally distributed over the grain boundaries, maximizing the grain boundary structure. Additionally, some Pr penetrates the region of the main phase grains, generating Pr_2_Fe_14_B, which partially compensates for the Br performance. Gallium (Ga) is incorporated in the diffusion source composition largely to lower the melting point and boost the diffusion efficiency. Some research shows [27] that a high-Ga, low-B composition in Nd-Fe-B contributes to producing Nd_6.1_Fe_13_Ga_1_ at the grain boundaries, which provides weak magnetic grain borders and strengthens decoupling effects while increasing grain boundary wettability.

**Figure 9:**
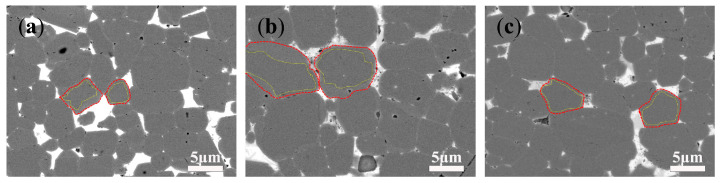
BSE-SEM images of the samples with a diffusion depth of 100 μm: (**a**) D1; (**b**) D2; (**c**) D3. Yellow circle marked is grain core, between red circle and yellow circle is grain shell.

Figure 9 shows the BSE-SEM images of the three samples after diffusion at a depth of 100 μm. In the D1 sample, a thick Tb-rich shell layer is found, with finer grains compared with the other samples, contributing to its higher performance over the D2 sample [28]. However, many triangular grain boundaries are also found in the D1 sample, which do not positively improve its performance [29]. These grain boundaries reduce the proportion of the main phase grains, preventing future performance improvement. The morphology of the D2 sample at 100 μm depth is similar to that at 50 μm depth, with a large quantity of Tb entering the main phase, resulting in a thick Tb-rich shell layer. While this boosts Hcj, it diminishes Br and BH(max), consuming extra Tb and decreasing its subsequent diffusion efficiency. In the D3 sample, several lighter-colored shell layers are equally dispersed around the main phase instead of deeply penetrating into it. Additionally, elongated white lines may be visible between the grains, indicating the presence of Nd-rich grain border phases that isolate the main phase grains. This arrangement limits the propagation of reverse magnetic domains formed by the demagnetizing field [30,31], enhancing Hcj and improving the Tb usage efficiency.

**Figure 10:**
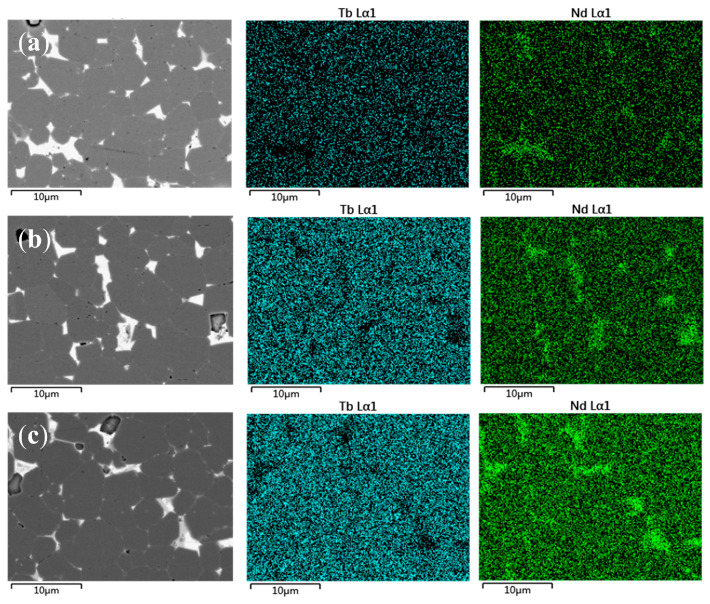
BSE-SEM images and EDS mapping of the samples with a diffusion depth of 300 μm: (**a**) D1; (**b**) D2; (**c**) D3.

To validate the diffusion depth of the various diffusion sources, EDS mapping was carried out at approximately 300 μm from the diffusion surface. The data, presented in Figure 10, suggest a considerable drop in the Tb concentration at this depth. As the diffusion depth rises, the Tb-rich shell structure gradually becomes thin and eventually disappears. This severely limits the further development of the magnet’s Hcj and constitutes the fundamental limitation of the GBD approach. Based on the statistical outcomes of the EDS surface scan, the apparent Tb concentrations are 5.07, 5.12, and 5.45 for D1, D2, and D3, respectively. The apparent Tb concentrations in D1 and D2 are pretty similar, while D3 has a considerably higher Tb concentration. This shows that low-melting-point, multi-component high-entropy diffusion sources can lead to an increase in the diffusion depth.

**Figure 11:**
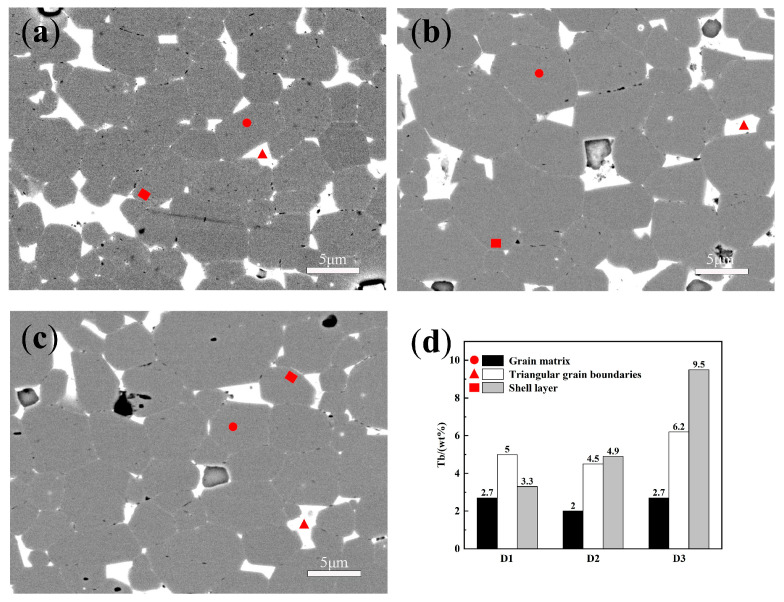
Comparison of EDS spot scan locations: (**a**) D1; (**b**) D2; (**c**) D3. (**d**) Tb content at a diffusion depth of 300 μm.

To further study the diffusion depth at 300 μm, point scans utilizing EDS were undertaken, as shown in Figure 11. The points were selected from within the main phase grains, the triangular grain borders, and the Tb-rich shell layers. The Tb concentrations at each point are displayed in Figure 11d. It is obvious that in the D3 sample, the matrix, triangular grain boundaries, and shell layers still retain considerable levels of Tb. Notably, the shell layer reveals a concentration of 9.5 wt.% Tb. This suggests that the D3 diffusion source demonstrates a deeper diffusion depth.

It is interesting that the BSE-SEM images and point scan data suggest the presence of two separate types of oxides, as shown in Figure 11. The first form is Fe-containing oxides, appearing as pure black blocks, largely made of Nd, Fe, and O. Additionally, Tb- and Nd-rich oxides are observed in the D2 and D3 samples. These oxides similarly appear as blocks but differ from the Fe-containing oxides by having dark edges and lighter cores. This discrepancy is due to the limited amount of Fe, with the principal constituents being rare earth elements and oxygen. The composition of these oxides is given in Table 4, with the Tb content higher than that in the shell structure. In the D2 sample, these oxides contain up to 22 wt.% Tb, while in the D3 sample, the amount is 16.7 wt.%. The high rare earth content in these oxides does not contribute to the magnetic performance. Instead, it depletes the heavy rare earth elements and diminishes the volume fraction of the main phase, adversely reducing the performance during manufacture. The D1 sample did not exhibit such rare earth oxides, but the high Tb content in the oxides of the D2 sample coincides with its relatively low performance.

During the diffusion process, it is vital to avoid the creation of such oxides. While oxidation during sample preparation and testing is tough to avoid, the presence of these rare-earth-rich oxides is detrimental to the magnetic characteristics. Efforts should be made to minimize their creation to increase performance.

## 4. Conclusions

This work explores the diffusion effects of Tb and Tb-containing alloys as diffusion sources on a Nd-Fe-B magnet. According to the results, the Hcj of the magnet at 20 °C grew dramatically by 49.4% after diffusion, reaching 27.7 kOe. We examined the benefits and drawbacks of Tb, Tb_60_Nd_5_Al_30_Ga_5_, and Tb_65_Pr_10_Nd_5_Al_5_Cu_10_Ga_5_ diffusion sources. The Tb diffusion source effectively boosts Hcj but results in excessive Tb entering the main phase, damaging Br and diminishing the use efficiency of Tb. The Tb_60_Nd_5_Al_30_Ga_5_ diffusion source demonstrates remarkable diffusion efficiency and can serve as a promising alternative for the GBD modification of thick magnets. Under the experimental conditions, Al and Tb elements significantly penetrate the main phase, lowering its quantity and severely impacting the magnetic characteristics. Therefore, diffusion sources with a high Al concentration require proper changes to the GBD heat treatment technique for maximum performance. The Tb_65_Pr_10_Nd_5_Al_5_Cu_10_Ga_5_ diffusion source exhibits the best overall performance in this experiment. It shows a thin layer of grid-like grain boundaries that are smooth and fine, decreasing magnetic coupling between the main phase grains. Tb produces a shell structure with a larger anisotropy field around the main phase grains, improving Hcj. This decreases Tb entrance into the main phase, hence increasing the diffusion depth and utilizing Tb more efficiently. Furthermore, Cu and Al improve the wetting of the grain boundary, which produces the optimal grain boundary structure. Future research on diffusion sources should focus more on scientific component design and examine optimum diffusion processes for diverse diffusion source compositions to further enhance the performance and stability of magnets.

## Figures and Tables

**Table 1:** Composition ratios of diffusion source slurry and Tb weight gain ratio.

	Tb Weight Gain Ratio (%)	Tb (wt.%)	Pr (wt.%)	Nd (wt.%)	Al (wt.%)	Cu (wt.%)	Ga (wt.%)
D1	0.8	100	/	/	/	/	/
D2	0.8	60	/	5	30	/	5
D3	0.8	65	10	5	5	10	5

**Table 3:** Br temperature coefficient α and Hcj temperature coefficient β of the magnets before and after diffusion.

	20–90 °C		20–140 °C	
	α (%/°C)	β (%/°C)	α (%/°C)	β (%/°C)
Original	−0.073	−0.645	−0.097	−0.560
D1	−0.089	−0.337	−0.099	−0.374
D2	−0.095	−0.395	−0.103	−0.411
D3	−0.090	−0.343	−0.098	−0.382

**Table 4:** Table of oxide point scanning data at a depth of 300 μm.

	Nd (wt.%)	Tb (wt.%)	Fe (wt.%)	O (wt.%)
Point 1	53.2	22.0	2.9	21.9
Point 2	57.0	16.7	3.8	22.5
Point 3	38.2	4.5	49.1	8.2

## Data Availability

The original contributions presented in this study are included in the article. Further inquiries can be directed to the corresponding author.
